# Investigation of differences in susceptibility of *Campylobacter jejuni* strains to UV light-emitting diode (UV-LED) technology

**DOI:** 10.1038/s41598-023-35315-0

**Published:** 2023-06-10

**Authors:** Arturo B. Soro, Daniel Ekhlas, Maitiú Marmion, Amalia G. M. Scannell, Paul Whyte, Declan J. Bolton, Catherine M. Burgess, Brijesh K. Tiwari

**Affiliations:** 1 Teagasc Food Research Centre, Ashtown, D15 DY05 Dublin Ireland; 2 UCD School of Veterinary Medicine, University College Dublin, Belfield, D04 V1W8 Ireland; 3 Infectious Diseases in Humans, Service Foodborne Pathogens, Sciensano, J. Wytsmanstraat 14, 1050 Brussels, Belgium; 4 UCD School of Agriculture and Food Science, University College Dublin, Belfield, D04 V1W8 Ireland; 5 UCD Centre for Food Safety, University College Dublin, Belfield, D04 V1W8 Ireland; 6 UCD Institute of Food and Health, University College Dublin, Belfield, D04 V1W8 Ireland; 7 Teagasc Food Research Centre, Ashtown, Dublin, D15 DY05 Ireland

**Keywords:** Microbiology, Antimicrobials, Applied microbiology, Bacteria, Biofilms, Pathogens

## Abstract

*Campylobacter jejuni* remains a high priority in public health worldwide. Ultraviolet light emitting-diode technology (UV-LED) is currently being explored to reduce *Campylobacter* levels in foods. However, challenges such as differences in species and strain susceptibilities, effects of repeated UV-treatments on the bacterial genome and the potential to promote antimicrobial cross-protection or induce biofilm formation have arisen. We investigated the susceptibility of eight *C. jejuni* clinical and farm isolates to UV-LED exposure. UV light at 280 nm induced different inactivation kinetics among strains, of which three showed reductions greater than 1.62 log CFU/mL, while one strain was particularly resistant to UV light with a maximum reduction of 0.39 log CFU/mL. However, inactivation was reduced by 0.46–1.03 log CFU/mL in these three strains and increased to 1.20 log CFU/mL in the resistant isolate after two repeated-UV cycles. Genomic changes related to UV light exposure were analysed using WGS. *C. jejuni* strains with altered phenotypic responses following UV exposure were also found to have changes in biofilm formation and susceptibility to ethanol and surface cleaners.

## Introduction

*Campylobacter* spp. are currently some of the most common foodborne pathogens and are estimated to be associated with approximately 500 million cases of campylobacteriosis cases each year worldwide. *Campylobacter jejuni* has been reported as the causative agent in most cases^1^. Poultry meat is often contaminated (60–80%) with *Campylobacter* spp. and is considered the principal source of infection in humans. Large-scale production of poultry has contributed to the spread of *C. jejuni* among flocks resulting in high levels of this bacterium in retail poultry meat. Consequently, efforts have been made at farm and processing levels to reduce the numbers of *C. jejuni* in poultry meat. Several interventions such as antimicrobial agents, vaccines, hot water, and steam treatment of carcasses have been studied as potential interventions in the poultry production chain^2^.

Ultraviolet (UV) light has emerged as a potential disinfection technology due to its effectiveness in the microbial decontamination of surfaces, water and air. Application of this non-thermal technology in liquid food and food surfaces has also been evaluated in the agri-food sector, with the aim of future implementation in the food chain^3^. UV light is situated in a specific wavelength range of 100–400 nm in the electromagnetic spectrum, where the region of 200–280 nm, also known as UV-C, has been shown to have the maximum inactivation effect for a wide range of microorganisms. The mechanisms of action of UV-C are well described in the literature^4,5^ and involve the formation of dimers in the DNA such as cyclobutane pyrimidine dimers (CPDs) and pyrimidine 6–4 pyrimidone photoproducts (6-4PPs) that result in lesions. These lesions interfere with RNA transcription and DNA replication, disrupting the normal function of the cell resulting in cell death^3,4^.

For the generation of UV light, mercury lamp devices are widely used in industry. However, mercury poses a toxic threat, which can have an impact on humans and the environment. Therefore, other alternatives, such as UV light-emitting diode (UV-LED) technology have emerged in recent years to overcome this issue. UV-LED devices present other advantages to mercury lamps such as low-cost, high durability, low heat and energy emissions and flexibility, among others. Despite this, these novel devices require further investigation for their potential implementation as disinfection strategies in the agri-food sector^5,6^.

According to Alvarez-Ordonez et al.^7^, novel processing technologies employed for food decontamination, like UV light, may trigger an adaptive response in some bacteria and lead to more persistent cells due to the characteristic sub-lethality of the stress. Thus, the effectiveness of disinfection may be reduced after several treatments with the same technology^7^. In particular, UV light treatments have been documented to be affected by the microbial growth rate, optical properties of the matrix and microbial strain and species differences. The latter may be of relevance when evaluating the effectiveness of UV light where high variability in UV resistance has been shown in strains of the same species^8^. For instance, Haughton et al.^9^ observed considerable variation in the inactivation kinetics of 10 *Campylobacter* strains when a UV lamp and UV-LED devices were applied. Another study also identified different susceptibilities among *Listeria monocytogenes* strains exposed to UV light but the growth phase did not influence susceptibility^10^. Evidence of strain-specific variability to resist UV light suggests that more information is required to understand this phenomenon to support future prospects for UV-LED implementation^11^. Other challenges exist regarding the use of this technology, including the effects of repeated UV treatment on the susceptibility of bacterial cells, biofilm formation and/or co-selection for decreased susceptibility to disinfectants. Although the association between biofilm formation and UV resistance in bacterial cells following UV exposure is underexplored, resistance to UV light has been shown to have cross-protective effects against stressors such as ethanol, acid, heat, and hydrogen peroxide. However, studies evaluating this cross-protection phenomenon are not sufficient to draw any firm conclusions^7,10–12^.

The objectives of this study were: (i) to investigate the susceptibility of eight *C. jejuni* field isolates and one reference strain to single exposure to UV-LED light (UV_280_), and four of the former strains to a repeated exposure to UV_280_ based on their observed UV susceptibility (ii) to examine any observed genomic alterations following treatment using whole-genome sequencing (WGS), (iii) to assess whether treatment with UV_280_ could result in increased resistance to sanitizing chemicals or induce biofilm formation.

## Results

### Susceptibility of *C. jejuni* strains to UV light

**Figure 1:**
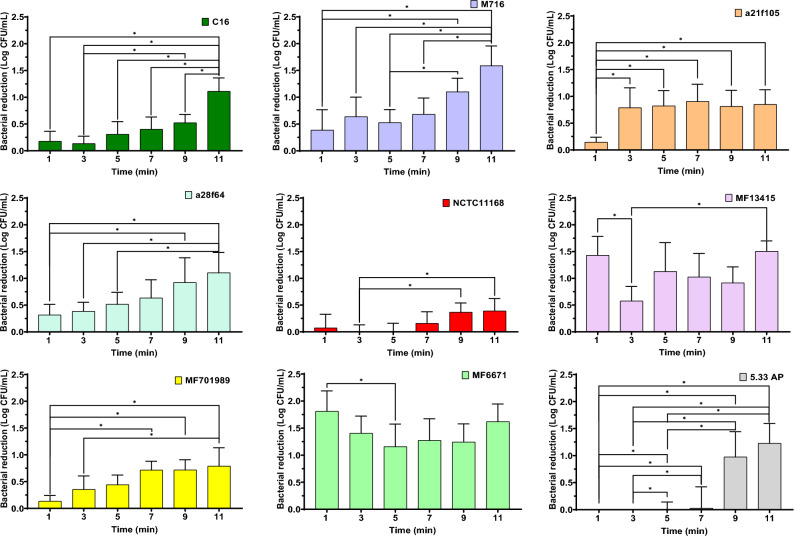
Bacterial reductions (Log CFU/mL) observed in the nine *C. jejuni* strains before and after UV exposure at 280 nm for 0, 1, 3, 7, 9 and 11 min. Statistical differences between treatments are indicated with * (p < 0.05).

The effects of exposure to UV_280_ for 1, 3, 5, 7 and 11 min on nine *C. jejuni* strains are presented in Fig. 1. In general, all the studied *C. jejuni* strains showed reductions greater than 0.8 Log CFU/mL after 11 min of UV light exposure, except for NCTC 11168, which was particularly resilient to the UV inactivation effect, with a maximum reduction of 0.39 ± 0.23 Log CFU/mL. MF6671 was the most susceptible strain to UV light exposure with the highest bacterial reductions (1.62 ± 0.33 Log CFU/mL), followed by MF716 (1.59 ± 0.37 Log CFU/mL) and MF13415 (1.51 ± 0.19 Log CFU/mL). Differences in inactivation kinetics were observed between these strains when UV at 280 nm was applied. Seven out of nine strains were resilient to longer UV light treatments such that a treatment period of 11 min did not result in significantly higher bacterial reductions than lower treatment times (p ≥ 0.05).

### Susceptibility of selected *C. jejuni* strains to single versus double UV treatments

**Figure 2:**
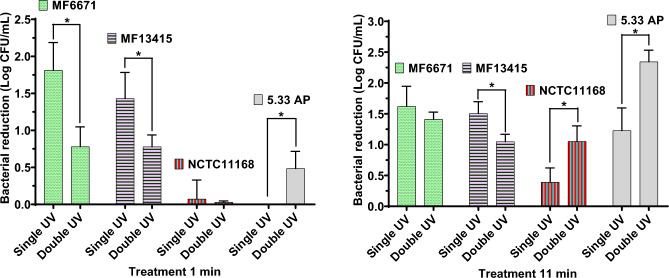
Bacterial reductions (Log CFU/mL) observed in the four selected *C. jejuni* strains when subjected to one or two cycles of UV at 280 nm for 1 and 11 min. Statistical differences between the treatments are indicated with * (p < 0.05).

MF6671, MF13415, 5.33 AP, and NCTC 11168 strains were selected due to their differing inactivation kinetics under UV light at 280 nm, as shown in Fig. 1. To evaluate the effect of repeated UV exposure on these strains, bacterial reductions after a double UV treatment were compared with reductions from single treatment (see Fig. 2). Surprisingly, the effectiveness of the second treatment with UV_280_ was significantly decreased when applied for 1 min to MF6671 (from 1.81 to 0.78 Log CFU/mL) and MF13415 (from 1.43 to 0.78 Log CFU/mL) strains, and 11 min to MF13415 (from 1.51 to 1.05 Log CFU/mL) (p < 0.05). Furthermore, the opposite effect was observed for 5.33 AP, with reductions that increased from 0 to 0.49 Log CFU/mL after 1 min exposure and 1.23–2.35 Log CFU/mL after 11 min exposure (p < 0.05). NCTC 11168 had an increase in bacterial reductions from 0.39 to 1.05 Log CFU/mL after 11 min exposure (p < 0.05). Thus, repeated treatment with UV_280_ significantly increased the susceptibility of the latter strains. Moreover, treatments of 11 min with UV_280_ were shown to reduce the tolerance of the *C. jejuni* strains treated for 2 UV-cycles than the ones treated for 1 min (p < 0.05).

### Whole-genome sequencing (WGS) analysis

**Figure 3:**
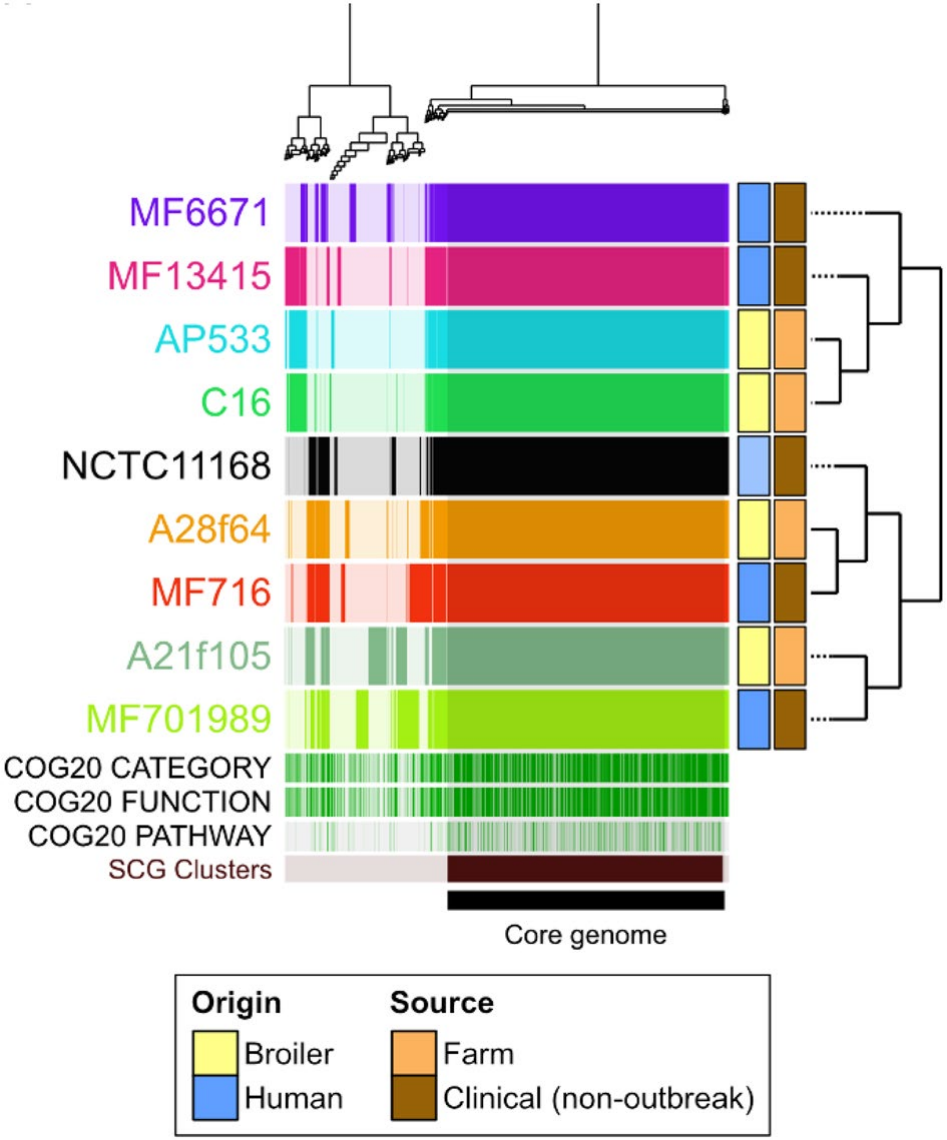
Cluster-tree analysis of pangenomes of *C. jejuni* strains and origin and source of the isolates included with nine strains not subjected to UV light.

WGS analysis was conducted in order to investigate potential differences in the genomes of the *C. jejuni* strains that displayed different inactivation kinetics before and after UV treatment. The pangenome of the 9 *C. jejuni* strains is presented in Fig. 3. In the core genome of these strains, 12,334 gene calls and 1356 gene clusters were found. Moreover, the total genome consisted of 2173 gene clusters and 15,414 genes. The pangenome of these strains displayed two clusters, of which MF6671, MF13415, 5.33 AP, and C16 strains were grouped together, and likewise NCTC 11168, A28f64, MF716, A21f105, and MF701989 strains. Origin and source of the isolated strains did not influence the clustering.

**Figure 4:**
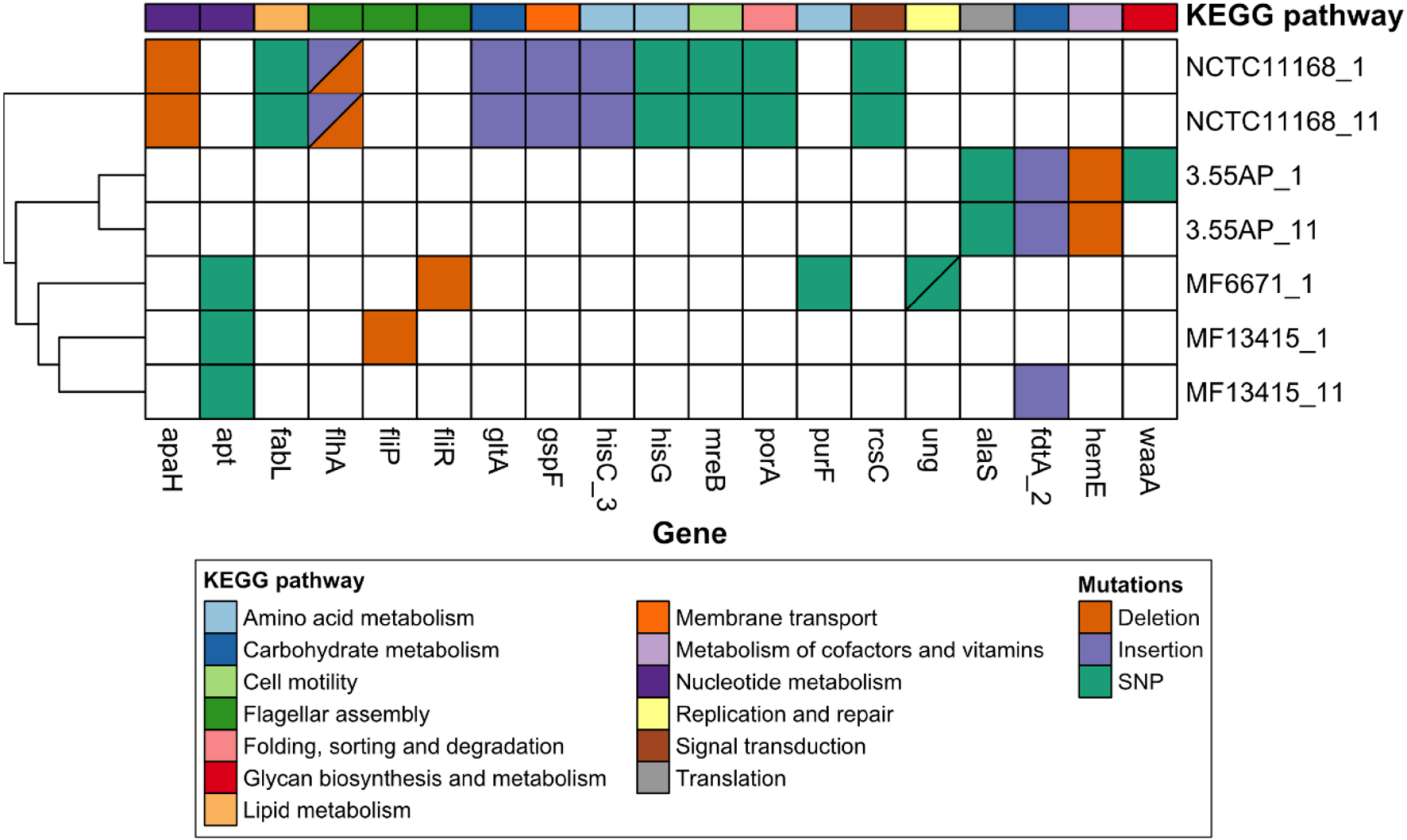
Heatmap of Snippy findings for selected *C. jejuni* strains subjected to UV at 280 nm for 1 and 11 min is presented, where the type of mutation: single nucleotide polymorphisms (SNPs), insertions and deletions; and KEGG pathways included for every mutated gene.

Potential mutations in the genome of UV-treated *C. jejuni* strains were analysed using Snippy to compare UV-treated genomes with non-treated ones. A heatmap of mutations based on SNPs and indels is presented in Fig. 4. Mutations in non-coding sequence (CDS) regions or those affecting hypothetical proteins were not considered and can be found in the Supplementary material (Supplementary Table S.1). In general, UV-induced mutations occurred regardless of the treatment time in the genome of NCTC 11168, 3.55 AP, and the *apt* gene in MF6671 and MF13415, encoding adenine phosphoribosyltransferase, when exposed to UV light at 280 nm for 1 or 11 min. Moreover, mutations due to SNPs, deletions, and insertions were equally observed. Each mutated gene was mapped to a KEGG pathway to investigate the potential impact of UV light on bacterial functioning and structures. NCTC 11168 was found to have the highest number of mutations, with a broad range of metabolic pathways, structures, and functions affected by UV exposure and with identical response, despite being subjected independently to different UV exposure times (Fig. 4). An SNP mutation in the *waaA* gene (glycan biosynthesis and metabolism) was found in the 5.33 AP strain exposed to UV for 1 min, unlike the same strain treated for 11 min. Furthermore, other mutations in genes associated with translation, and carbohydrate and cofactors and vitamins metabolism were detected when 5.33 AP was subjected to both exposure times. After UV exposure, the *apt* gene involved in nucleotide metabolism presented a mutation in MF6671 and MF13415 strains. Other mutations in genes related with carbohydrate and amino acid metabolism, flagellar assembly, and replication and repair were found in these two strains.

### Assessment of biofilm formation in *C. jejuni* strains

The growth of *Campylobacter* biofilms was assessed in nutrient-rich (TSB) and nutrient-poor (M9) media under both aerobic and microaerobic conditions at two different temperatures (37 and 4 °C), and the results were assessed after 24 h of incubation. This protocol was repeated with cultures exposed to UV_280_ for 7 min. The strongest biofilm formation was observed in each *C. jejuni* isolate when grown in a nutrient-rich medium at 37 °C under microaerobic conditions and results are summarised in Table 1. Of the 9 isolates, three of them showed an ability to form strong biofilms at low temperatures in the absence of environmental oxygen concentrations, while low nutrient availability contributed to the formation of weaker biofilms in most isolates. However, the reference *C. jejuni* strain NCTC 11168 and the isolates C16, MF701989, MF13415, MF6671, 5.33 AP, and a28f64 showed some moderate biofilm formation at 37 °C under microaerobic conditions in nutrient-limited (M9) medium. Under aerobic conditions at 4 °C, stronger biofilm formation was observed in the rich media in only one isolate (a21f105), while moderate biofilm formation was observed in two further isolates (C16 and MF701989). Under the same conditions, weak biofilm formation was observed in most *Campylobacter* isolates in low nutrient abundance.

**Table 1:** Biofilm formation by *Campylobacter jejuni* isolates under varied conditions of temperature, oxygen abundance and nutrient availability before and after UV light treatment.

Treatment	37 °C Aerobic M9		37 °C Aerobic TSB	
	Untreated	Treated	Untreated	Treated
a21f105	**++**	**−**	**++**	**+**
C16	**+**	**−**	**++**	**+**
MF701989	**+**	**−**	**++**	**+**
MF716	**−**	**−**	**++**	**+**
MF13415	**+**	**+**	**+**	**+**
MF6671	**−**	**+**	**+**	**++**
5.33 AP	**+**	**−**	**++**	**+**
a28f64	**−**	**+**	**−**	**+**
NCTC 11168	**−**	**+**	**+**	**+**

Treatment	37 °C Microaerobic M9		37 °C Microaerobic TSB	
	Untreated	Treated	Untreated	Treated
a21f105	**++**	**+**	**+++++**	**+**
C16	**++**	**−**	**+++++**	**+**
MF701989	**++**	**−**	**+++++**	**+**
MF716	**+**	**−**	**+++**	**+**
MF13415	**++**	**+**	**++++**	**+**
MF6671	**+++**	**+**	**+++++**	**+**
5.33 AP	**+++**	**−**	**+++++**	**+**
a28f64	**+++**	**−**	**+++++**	**+**
NCTC 11168	**+++++**	**+**	**+++++**	**+**

Treatment	4 °C Aerobic M9		4 °C Aerobic TSB	
	Untreated	Treated	Untreated	Treated
a21f105	**++**	**+**	**+++++**	**+**
C16	**++**	**−**	**++++**	**+**
MF701989	**++**	**−**	**++++**	**+**
MF716	**+**	**−**	**+**	**+**
MF13415	**+**	**−**	**+**	**−**
MF6671	**−**	**−**	**+**	**+**
5.33 AP	**−**	**−**	**+**	**−**
a28f64	**−**	**−**	**−**	**+**
NCTC 11168	**−**	**−**	**+**	**+**

Treatment	4 °C Microaerobic M9		4 °C Microaerobic TSB	
	Untreated	Treated	Untreated	Treated
a21f105	**−**	**+**	**+**	**+**
C16	**+**	**−**	**+**	**+**
MF701989	**+**	**−**	**+**	**−**
MF716	**−**	**−**	**+**	**−**
MF13415	**+**	**−**	**++**	**−**
MF6671	**−**	**−**	**+**	**−**
5.33 AP	**−**	**−**	**−**	**−**
a28f64	**−**	**−**	**−**	**−**
NCTC 11168	**−**	**−**	**−**	**+**

The parameters for the strength of biofilm formation were based on the logical test: X > 1,"+++++", X > 0.8, "++++", X > 0.6, "+++", X > 0.3, "++", X > 0.1, "+", H90 < 0.1, "−", where X is the optical density (OD) at 600 nm.

Isolates treated with UV light showed a reduced ability to form biofilms compared to untreated isolates across each of the conditions used in this study. Growth conditions with abundant nutrients (TSB), warmer temperatures (37 °C) and the presence of oxygen resulted in the strongest biofilm growth in UV_280_ treated cells, although biofilm formation capacity was still less in untreated cells for all strains, with the exception of isolates a28f64 and MF6671. Low growth temperature and low nutrient abundance (M9 medium) resulted in significant reductions (p < 0.05) in biofilm formation after UV treatment for all isolates, with the exception of a21f105. Treatment with UV_280_ significantly reduced (p < 0.05) biofilm formation under all conditions for each isolate investigated (Table 1).

### Assessment of resistance of *C. jejuni* isolates to sanitizers

In Table 2, 3 out of 9 strains showed susceptibility to ethanol, domestic bleach (sodium hypochlorite), and domestic surface cleaner solutions prior to UV treatment of cell suspensions, with the exception of C16, 5.33 AP, NCTC 11168. Moreover, the antimicrobial effect of these solutions was reduced in the majority of *C. jejuni* strains when exposed at 4 °C. While strains MF13415 and NCTC 11168 showed no cell activity at 4 °C, other strains including A21F105, C16, MF701989, MF716, and a28f64 showed higher resilience against the compounds studied, even at recommended working concentrations. The application of UV-LED technology improved or maintained the inactivation efficacy of these disinfectants in 5 of 9 strains. Nevertheless, isolate C16 at 42 °C showed reduced sensitivity to ethanol following UV treatment, while only the surfactant-based cleaner still showed an effect against UV-treated MF716 at 42 °C. Similarly, resistance to EtOH and NaOCl-based cleaners was higher in UV treated cells of MF6671 and in MF701989 to EtOH and the surface cleaner (Table 2). Increased susceptibility to EtOH in particular was also seen after UV treatment in isolates MF13415, NCTC 11168, and especially 5.33 AP, which was more susceptible to each class of antimicrobial tested. This is notable as untreated suspensions of 5.33 AP showed greater resilience when disinfecting agents were employed. Exposure to UV light and incubation at 4 °C resulted in increased susceptibility of a21f105 to ethanol, and MF701989 and 5.33 AP to NaOCl, but at the same time MF701989 showed increased resilience to the surface cleaner and MF6676 to all the evaluated solutions (Table 2). MF13415 and NCTC 11168 wild types, interestingly, showed no survival at 4 °C, but mutated strains were able to survive at this temperature.

**Table 2:** Efficacy of common sanitizers against *Campylobacter jejuni* isolates. W indicates effect (total reduction) at recommended working concentration. 0.5W indicates effect at half of working concentration. NE indicates No Effect, NG indicates No Growth before biocide exposure.

Strain	Temp	42 °C			4 °C		
		EtOH	NaOCl	Surface cleaner	EtOH	NaOCl	Surface cleaner
a21f105	Untreated	0.5W	W	W	W	NE	NE
	Treated	0.5W	W	W	0.5W^a^	NE	NE
C16	Untreated	W	NE	0.5W	NE	NE	0.5W
	Treated	NE^b^	NE	0.5W	NE	NE	0.5W
MF701989	Untreated	0.5W	W	0.5W	W	NE	W
	Treated	W^b^	W	W^b^	W	W^a^	NE^b^
MF716	Untreated	0.5W	W	0.5W	W	NE	NE
	Treated	W^b^	NE^b^	0.5W	NG	NG	NG
MF13415	Untreated	W	W	0.5W	NG	NG	NG
	Treated	0.5W^a^	W	0.5W	0.5W	W	0.5W
MF6671	Untreated	0.5W	0.5W	0.5W	0.5W	0.5W	0.5W
	Treated	W^b^	W^b^	0.5W	W^b^	NE^b^	W^b^
5.33 AP	Untreated	NE	NE	W	NE	NE	W
	Treated	0.5W^a^	0.5W^a^	0.5W^a^	NE	W^a^	W
a28f64	Untreated	W	0.5W	0.5W	W	W	NE
	Treated	0.5W^a^	0.5W	0.5W	W	W	0.5W^a^
NCTC 11168	Untreated	W	NE	0.5W	NG	NG	NG
	Treated	0.5W^a^	NE	0.5W	NE	NE	W

^a^Indicates improved effectiveness after treatment.

^b^Indicates reduced effectiveness.

## Discussion

The application of UV-LED technology to reduce *Campylobacter* numbers in liquid, surfaces, and food has been investigated previously in other studies^13–15^. However, to the best of the authors’ knowledge, differences in bacterial inactivation kinetics after UV treatment have only been evaluated by Haughton et al.^14^, Haughton et al.^9^, and in the current study. The former study observed that different susceptibilities among *Campylobacter* isolates towards UV light at 395 nm in a transparent medium was a result of the biological effect and not of any factor of light intensity attenuation^14^. To evaluate the susceptibility of *Campylobacter* to UV-LED in our study and compare strain susceptibilities, it was necessary to modify the absorbance of the medium in order to reduce UV light penetrability and the high decontamination effectiveness of the UV-LED technology in transparent liquid media. Compared to Haughton et al.^14^, the achieved bacterial reductions in the present study were 6 log lower (Fig. 1). This may be a consequence of reducing the penetrability of the UV light in the medium which may protect bacterial cells and favour their survival^13^. In the study of Haughton et al.^9^, *C. jejuni* suspensions in a mixture of MRD and UHT skim milk were treated with a UV lamp device at 254 nm and reductions of up to 6 log CFU/mL were observed for all 10 *Campylobacter* isolates, with a reduction of 3.5 log CFU/mL observed for the least susceptible strain. Although reductions were lower in our study (≤ 1.6 log CFU/mL), probably due to a higher fat content (2%) in the milk matrix or the difference in UV wavelengths, differences in inactivation were also observed in all the studied strains after UV exposure. Pangenome analysis of the 9 *C. jejuni* strains resulted in two clusters independent of source and origin of isolates. Other authors, such as Thépault et al.^16^, Wilson et al.^17^, and Méric et al.^18^ investigated the pangenome of *C. jejuni* isolates with the aim of correlating their origin and source. They found this task challenging due to the high level of genotypic diversity.

The most noteworthy strains based on observed high variations in inactivation kinetics were selected and subjected to two cycles of UV light treatment at 280 nm. Interestingly, the repeated treatments with UV light at 280 nm had the opposite effect on *C. jejuni* reductions in the studied strains compared to UV single treatments. Thus, more susceptible strains after single UV treatments had an increased resistance after two UV cycles and vice versa. Álvarez-Molina et al.^21^ investigated the adaptation process of *Escherichia coli*, *Salmonella* spp. and *Listeria monocytogenes* to UV light after 10 repeated UV-cycles and observed that bacterial cells were more resilient to UV light afterwards. These authors suggested that this phenomenon may be a consequence of adaptive mutagenesis when cells are subjected to sub-lethal stress^19^. Although similar observations were made for MF6671 and MF13415 after two UV cycles, the increased susceptibility to UV light found in NCTC 11168 and 5.33 AP strains is in contrast with the former. It is important to note that MF6671 and MF13415 were the most susceptible strains to UV light and 5.33 AP and NCTC 11168 were the most resilient strains when subjected to a single UV treatment. Therefore, a correlation between both effects may be possible. However, insufficient information is currently available in the scientific literature to reach any conclusions. Dissimilarities detected in the alignments of UV-treated isolates may have resulted from induced missense mutations in the bacterial genome. To verify this, Snippy analysis was conducted comparing the genome of UV-treated with non-treated strains. Strains NCTC 11168 and 3.55 AP, which were more susceptible after two UV cycles, presented mutations in genes associated with signal transduction and translation. In contrast, mutations in genes *flip* and *fliR* (encoding for flagellar biosynthetic proteins FliP and FliR) were observed in strains which were more resilient to UV light after two UV cycles and are linked to motility and host colonisation^20^. These authors suggested that these reversible mutations are an adaptive mechanism to maintain genome stability (genome robustness) in *Campylobacter* spp. in response to stress factors like UV^20^. Mutation in the *fdtA* gene (encoding for TDP-4-oxo-6-deoxy-alpha-d-glucose-3,4-oxoisomerase) was also identified in our study, which has been linked to adhesion and colonisation of *E. coli*^21^. However, the function of this gene was not described in *C. jejuni* before. Furthermore, mutations in *purF* (encoding for amidophosphoribosyltransferase) and *apt* genes found in the least susceptible strains of this study are evidenced to be associated with a novel adaptive mechanism of *C. jejuni* to increase its probability of survival, based on promoting the genetic heterogeneity of the bacterial population^22^. Lastly, a mutation in *ung* gene, encoding for uracil-DNA glycosylase, observed in this study was also previously detected in other studies^23,24^. Although this gene was associated with initiation of base excision repair (BER) pathway, mechanisms induced by UV stress, Gaasbeek et al.^24^ and Dai et al.^23^ concluded that the mutation of this gene does not promote the repair of DNA damage or recombinational repair in *C. jejuni*. Thus, *C. jejuni* strains that were more resilient to UV presented mutations linked to survival mechanisms.

For the biofilm formation analysis conducted in this study, variations in biofilm strength and presence/absence were observed in non-treated *C. jejuni* strains for the different conditions studied (4 and 37 °C; aerobic and microaerobic; rich and poor-nutrient media). Strain variability in biofilm formation has already been observed for other foodborne pathogens^25^. These authors indicated that further research is required to evaluate the bacterial biofilm formation under more realistic conditions^25^. Thus, our study demonstrated strain variability of *C. jejuni* in biofilm formation, even under cool, microaerobic, or poor-nutrient environments. In general, UV light diminished biofilm production in most of the studied strains, with greater reductions observed in the presence of additional stresses (4 °C and poor nutrient medium). Some of the previously mentioned mutated genes (*flhA*, *rcsC*, *mreB* and *waaA*) in NCTC 11168 and 5.33 AP may be associated with biofilm formation, since they are associated with cell motility, morphology and peptidoglycan formation^26–29^. According to Luo et al.^30^, UV light technology has been mainly assessed for inactivation of microorganisms already within formed biofilms. Nevertheless, there are a few studies investigating UV light as a treatment to prevent biofilm formation^30^. Studies investigating the use of UV to prevent biofilm formation by *E. coli* and *Pseudomonas aeruginosa* cells were successful in this regard^31–33^. However, the disruptive effect of UV light on the biofilm formation process may not last long^34^. Bacterial cultures in the present study were incubated for only 24 h and therefore, further investigation is required to assess this concern.

The antimicrobial activity of EtOH and selected surface cleaners was reduced when bacteria were subjected to low temperatures. This temperature-dependent activity of biocides has been extensively demonstrated for the majority of domestic and industrial surface cleaners by several authors^35^. In concordance with our study, Bakht et al.^36^ observed variations in antimicrobial susceptibility to biocides, such as EtOH 70% and NaOCl 5% in 120 *P. aeruginosa* strains, of which 59 were resilient to EtOH 8.75% and 33 strains to NaOCl 0.08%. In the present study, EtOH 70% and NaOCl 2% did not inactivate the 5.33 AP *C. jejuni* strain at 42 ℃. At the same time, C16 and NCTC 11168 were resilient to a 2% concentration of NaOCl under the same conditions. A treatment with UV light prior biocide exposure showed to improve or maintained the antimicrobial effectiveness of the selected biocides in the majority of the strains. The combined inhibitory effect of UV light together with EtOH or NaOCl has been observed for other pathogenic bacteria including *E. coli*, *Bacillus cereus*, *Cronobacter sakazakii*, *S.* Typhimurium, among others^37–39^. However, 4 *C. jejuni* strains showed increased tolerance to at least one of the studied biocides after UV treatment in the present study. The presence of mutated genes *apt* and *purF* in MF6671 which was identified as a tolerant strain to EtOH and NaOCl-based cleaners after UV exposure may increase the stress tolerance of these bacteria. Thus, *C. jejuni* cells with mutated purine biosynthesis genes (*purF* and *apt*) were more tolerant to hyperosmotic stress. This phenomenon may be caused by a cross-protection mechanism which could develop due to the mutagenic nature of UV light^40^. Hartke et al.^41^ studied the effect of pre-treatment with 254 nm UV on *Lactococcus lactis* and found that bacterial cultures increased tolerance to 20% (v/v) ethanol, heat (52 °C) and H_2_O_2_ (15 mM). These authors suggested that an overlapping regulation pathway between UV and other stresses may be ocurring^41^. To our best knowledge, studies, which found a cross-protection of UV light towards common industrial and domestic biocides like EtOH and NaOCl, are lacking. Future research should focus on comparing the findings of this study with transcriptomic analysis in order to better understand the effects of UV light on the bacterial genome, induced mutations, and its linkage with cross-protection.

## Methods

### *Campylobacter jejuni* strain selection and preparation

A total of 8 field isolates of *C. jejuni* and a reference strain (NCTC 11168) were obtained from the microbiology culture collection at Teagasc Food Research Centre, Ashtown (Dublin, Ireland). Strains were isolated from different sources (clinical and farm origin) as shown in the extended data section and were stored in defibrinated horse blood (Cruinn diagnostics limited, Dublin, Ireland) at − 80 °C. Preparation of isolates consisted of the resuscitation of bacterial stocks on Mueller Hinton agar plates (Oxoid, Basingstoke, UK) and subsequent incubation at 42 °C for 48 h under a microaerobic atmosphere. Colonies were streaked onto modified charcoal cefoperazone deoxycholate agar (mCCDA, Oxoid, Basingstoke, UK) supplemented with *Campylobacter* growth supplement (Oxoid, Basingstoke, UK). After incubating microaerobically for 48 h at 42 °C, isolated colonies were inoculated into 30 mL of Mueller Hinton broth (MHB) (Oxoid, Basingstoke, UK) and suspensions were incubated for 48 h at 42 °C in microaerobic conditions.

### Sample preparation and UV light treatments

Bacterial cell suspensions were centrifuged at 4000×*g* for 15 min and washed twice with 30 mL of maximum recovery diluent (MRD, Oxoid, Basingstoke, UK). A final *C. jejuni* concentration of ~ 5 log CFU/mL was achieved in 20 mL of a ultra-high treatment (UHT) milk (2% fat) diluted in MRD (1:4, v:v) in order to reduce the UV light penetrability in the medium and assist the survival of the cells^9^. Bacterial suspensions (20 mL) were poured into Petri dishes with a liquid depth of ~ 6 mm and volume capacity of 24 cm^3^ (height: 1.3 cm and diameter: 5.8 cm) and placed centrally in the LED chamber at a distance of 5 cm from the light source. Bacterial suspensions were treated with a UV-LED device (PearlLab Beam, Aquisense technologies, NC, USA) at a wavelength of 280 nm for 1, 3, 5, 7, 9 and 11 min. An in-depth description of the UV-LED device is provided and the wavelength of 280 nm was selected for its high inactivation effect^15,42^. Non-treated samples acted as controls. The UV_280_ dose was calculated by multiplying the measured fluence rate of the light (W/cm^2^) with the treatment time in min. The UV light fluence rate was measured using a radiometer (Opticalmeter, model ILT2400, International light technologies, MA, USA) and confirmed as 0.041 W/cm^2^. UV light doses for every treatment time are provided in the extended data section.

### *Campylobacter* enumeration

Immediately after UV treatment, *C. jejuni* levels were determined in the suspensions of UV-treated and control samples for each strain. Serial tenfold dilutions in MRD were prepared and 0.1-mL aliquots were plated onto mCCDA plates. After incubation for 48 h at 42 °C in microaerobic conditions, bacterial colonies were enumerated and the average counts of treated and control samples were determined. Bacterial reductions were calculated by subtracting the treated sample *C. jejuni* counts from the non-treated expressed in Log CFU units per mL of suspension.

### Repeated treatments of UV light on selected *C. jejuni* strains

Four *C. jejuni* strains were selected for further analysis due to their differing susceptibility to UV light: strains MF6671, MF13415, NCTC 11168, and 5.33 AP. Strain suspensions were prepared as detailed above, with 1 mL suspension inoculated into 9 mL of MRD. These ~ 5 log CFU/mL suspensions were treated with the UV_280_ for 6 s at a distance of 5 cm from the source in order to reduce the total *Campylobacter* population of all tested strains by 48–61% (enumerated as above). Surviving colonies following UV_280_ treatment were cultured in MHB and incubated microaerobically for 48 h at 42 °C. After incubation, suspensions were inoculated in a mixture of MRD and UHT milk, as detailed above. In this case, exposure times of 1 and 11 min with UV_280_ were selected to treat milk and MRD suspensions because of their different kinetics of inactivation. Non-treated samples served as controls. Enumeration of the survivors of the four *C. jejuni* strains was carried out for all the treatment and control samples with the previously described procedure.

### DNA extraction, whole-genome sequencing (WGS) and bioinformatics analysis

DNA extraction of MF6671, MF13415, NCTC 11168, and 5.33 AP strains was conducted from colonies that grew on mCDDA plated following exposure to UV_280_ for 1 and 11 min using the DNeasy^®^ Blood & Tissue kit (Qiagen, Manchester, UK) following the manufacturer’s instructions. Purity of extracted DNA was assessed using a NanodropTM 1000 Spectrophotometer (ThermoFisher Scientific, Eaton Socon, UK) and DNA concentrations were determined using a Qubit 4.0 Fluorometer (Invitrogen, ThermoFisher Scientific, Eaton Socon, UK). Sequencing was undertaken at the sequencing centre at Teagasc Food Research Centre Moorepark (Fermoy, Ireland). Preparation of DNA libraries was carried out with the Illumina DNA prep kit (Illumina, San Diego, CA, USA) following the manufacturer’s instructions. Subsequently, sequencing by 2 × 150 bp was performed using the NextSeq 2000 system (Illumina, San Diego, CA, USA) with P2 reagents.

Raw sequences were obtained for each strain tested. Raw sequences of the test strains not treated with UV_280_ were obtained from a previous study conducted by Truccollo et al.^43^ and the raw sequence of *C. jejuni* NCTC 11168 was recovered from the NCBI database (BioProject PRJNA8). Cleaning was a systemic process conducted using Trimmomatic (v0.3.8) after removing adapters, reads containing more than 10% undetermined bases (N > 10%) and low quality reads with a Qscore below or equal to 5 (Qscore ≤ 5) in a 50% of the total bases were removed. After cleaning, the quality of the reads was evaluated with FastQC (v0.11.8) in combination with MultiQC (v1.9) programs^44,45^. Before proceeding any further, the identification of the strains was carried out with Kraken 2 (v2.0.7 beta) through the standard Kraken 2 database^46^. Assembly of reads to contigs and scaffolds was performed using SPAdes (v3.13.0) with the –careful option. The quality of scaffolds was assessed through QUAST (v5.1.0) and MultiQC^45,47,48^. To visualise the pangenome of the studied *C. jejuni* strains, anvi’o (v7.1) workflow was employed in the obtained assembled scaffold.fasta files of non-treated and treated strains (https://merenlab.org/2016/11/08/pangenomics-v2/; accessed on 23 November 2022)^49^. The former files were converted into anvi’o contig databases through the ‘anvi-gen-contigs-database’ program. After this step, identification of genes in scaffolds was performed with Prodigal in order to detect open reading frames and their annotation was conducted using the NCBI’s Clusters of Orthologous Groups database (‘anvi-run-ncbi-cogs’ program)^50,51^ towards four HMM profiles of anvi’o provided by hidden Markov models (‘anvi-run-hmms’ program). In order to build the pangenome, similarities of the amino acid sequence were determined and compared within all genomes with NCBI blastp. Minbit heuristics of 0.5^52^ were employed to eliminate weak matches between amino acid sequences and the MCL algorithm (‘anvi-pan-genome’ program)^53^ was used to identify clusters.

Genomes of UV-treated strains were compared with non-treated strains using Snippy (v4.3.6) which establishes differences based on single nucleotide polymorphisms (SNPs) and small insertions and deletions (indels), also known as “variant calling”^54^.

### Biofilm assessment

*C. jejuni* isolates were grown overnight in Brain–Heart Infusion broth with 0.5% (v/v) defibrinated horse blood. A previously assessed treatment of UV_280_ was used to expose these bacterial suspensions for 7 min. Subsequently, these suspensions were used for the biofilm formation assay. Non-treated samples served as controls. The isolates were aliquoted into Eppendorf tubes and centrifuged at 13,000×*g* for 5 min. The supernatant was removed, and the pellet was washed in sterile ringer medium (Oxoid, Ltd., Basingstoke, UK). This was again centrifuged, and the supernatant discarded. The pellet was resuspended in Tryptic Soya broth (Oxoid, Ltd., Basingstoke, UK) and M9 medium (MP Biomedicals Germany LLC., Eschwege, Germany) and added in duplicates of 200 µL to a sterile 96 well plate. Four identical plates were prepared and each incubated under one of four conditions: 37 °C under environmental oxygen concentrations, 37 °C under microaerobic conditions, 4 °C under environmental oxygen concentrations, and 4 °C under microaerobic conditions. After 24 h, the medium was removed, and biofilms formed were analysed using a crystal violet staining protocol^55^. The parameters for the strength of biofilm formation were based on the logical test: X > 1,"+++++", X > 0.8, "++++", X > 0.6, "+++", X > 0.3, "++", X > 0.1, "+", H90 < 0.1, "−", where X is the optical density (OD) at 600 nm.

### Biocide susceptibility assessment

*Campylobacter jejuni* isolates were grown overnight in Brain–Heart Infusion broth with 0.5% (v/v) defibrinated horse blood. Suspensions were treated with UV_280_ for 7 min and control samples were non-treated. Antimicrobial resistance assessment was conducted following Balouiri et al.^56^. Briefly, bacterial suspensions grown overnight in Muller-Hinton Broth were diluted to an OD_600_ = 0.1 and subsequently, 1 ml of this suspension was used to inoculate molten Mueller Hinton agar at 45 °C (5% defibrinated horse blood in Mueller Hinton agar). Once set, 10 µl aliquots of solutions containing the working concentrations of common industrial sanitizing compounds including 70% (v/v) ethanol (EtOH) (Sigma-Aldrich Ltd., Arklow, Ireland), domestic bleach (< 5% chlorine based bleaching agents, 2% sodium hypochlorite) (Milton^®^, Proctor & Gamble, U.S.A), and domestic surface cleaner (5-chloro-2-methyl-4-isothiazolin-3-one and 2-methyl-2H-isothiazol-3-one) (2Work Multi-Surface Cleaner, 2Work Supplies, Sheffield, UK) and 50% of these concentrations were added to the plate with the purpose of mimicking dilutions events in an industrial or home setting. After 48 h growth at 42 °C under aerobic and microaerobic conditions, bacterial growth in the presence of these antimicrobial compounds was assessed^56^.

### Statistical analysis and visualisation

UV light treatments were conducted in duplicate and three independent experiments were assessed (N = 6). Normality of the data was tested using Kolmogorov–Smirnov test and comparison of treated and control samples was conducted through factorial analysis of variance (ANOVA) for each of the *C. jejuni* strains. Statistical differences were obtained using the Tukey post hoc test at a α < 0.05 level. The GraphPad Prism program (GraphPad Prism version 8.4.2 Inc, San Diego, CA, USA) was employed to perform the statistical analysis and create the presented graphs. Pangenome visualisation was performed and edited with the anvi’o interactive interface and the program ‘anvi-display-pan’. Snippy findings were visualized using R (v4.2.1; 2022-06-23) and RStudio (v2021.09.2) together with the pheatmap package (v1.0.12). The quality of pangenome and snippy figures was improved using Affinity Designer (v1.8.5.703, Serif).

## Supplementary Information

Supplementary Information 1.

Supplementary Information 2.

## Data Availability

Raw sequenced data of the 8 *C. jejuni* isolates were obtained from the dataset of BioProject ID PRJNA688841 and raw dataset of UV-treated isolates from this study can be found under BioProject ID PRJNA906059.
