# Supplementary material for: Investigation of differences in susceptibility of Campylobacter jejuni strains to UV light-emitting diode (UV-LED) technology
Source: Sci Rep. 2023 Jun 10;13:9459. doi: 10.1038/s41598-023-35315-0 (PMC10257703; doi:10.1038/s41598-023-35315-0)
Supplement: Supplementary file 1 — Supplementary Information 1. [file 41598_2023_35315_MOESM1_ESM.docx]

**Supplementary material**

**Table 1.** *Campylobacter jejuni* isolates from farm and clinical source selected for this study.

| **Isolate ID** | **Origin** | **Source** | **Year isolated** | |
| --- | --- | --- | --- | --- |
| a21f105 | Broiler | Farm | 2018 |  |
| a28f64 | Broiler | Farm | 2018 |  |
| C16 | Broiler | Farm | 2008 |  |
| 5.33AP | Broiler | Farm | 2008 |  |
| MF13415 | Human | Non-outbreak clinical | 2016 |  |
| MF716 | Human | Non-outbreak clinical | 2016 |  |
| MF6671 | Human | Non-outbreak clinical | 2016 |  |
| MF701989 | Human | Non-outbreak clinical | 2017 |  |
| NCTC 11168 | Human | Non-outbreak clinical | 2021 |  |

**Table 2.** Final UV light doses (W x min x cm^-2^) calculated for each treatment time (min) when the UV light fluence rate (W/cm^2^) was measured at a wavelength of 280 nm.

|  | **UV light Dose (W x min x cm^-2^)**  **Treatment time (min)** | | | | | |  |
| --- | --- | --- | --- | --- | --- | --- | --- |
|  | **Fluence rate (W/cm^2^)** | **1** | **3** | **5** | **7** | **9** | **11** |
| **Wavelength**  **280 nm** | 0.041 | 0.041 | 0.123 | 0.205 | 0.287 | 0.369 | 0.451 |
